# Soluble Vascular Adhesion Protein 1 (sVAP-1) as a biomarker for pregnancy complications: A pilot study

**DOI:** 10.1371/journal.pone.0284412

**Published:** 2023-05-30

**Authors:** Marianna Danielli, Roisin C. Thomas, Clare L. Gillies, David G. Lambert, Kamlesh Khunti, Bee Kang Tan

**Affiliations:** 1 Department of Cardiovascular Sciences, University of Leicester, Leicester, United Kingdom; 2 Diabetes Research Centre, Leicester General Hospital, Leicester, United Kingdom; 3 National Institute for Health and Care Research (NIHR) Applied Research Collaboration–East Midlands (ARC-EM), Leicester General Hospital, Leicester, United Kingdom; Rivers State University Teaching Hospital, NIGERIA

## Abstract

**Background:**

Vascular adhesion protein 1 (VAP-1) has been implicated in a wide range of clinical conditions. Moreover, serum levels are associated with disease prediction and progression in several clinical studies. There is a paucity of data on VAP-1 and pregnancy. Given the emerging role of VAP-1 in pregnancy, the aim of this study was to examine sVAP-1 as an early biomarker of pregnancy complications, especially hypertension during pregnancy. The objectives of the study are to associate sVAP-1 levels with other pregnancy complications, patient demographics and blood tests performed throughout pregnancy.

**Methods:**

We conducted a pilot study in a cohort of pregnant women (gestational week lower than 20 at the time of recruitment) attending their first antenatal ultrasound scan at the Leicester Royal Infirmary (LRI, UK). Data were both prospectively generated (from blood sample analysis) and retrospectively collected (from hospital records).

**Results:**

From July and October 2021, a total of 91 participants were enrolled. Using ELISA (enzyme-linked immunosorbent assay), we found reduced serum levels of sVAP-1 in pregnant women with either pregnancy induced hypertension (PIH) (310 ng/mL) or GDM (366.73 ng/mL) as compared to controls (427.44 ng/mL and 428.34 ng/mL, respectively). No significant difference was found between women with FGR compared to controls (424.32 ng/mL vs 424.52 ng/mL), and patients with any pregnancy complications compared to healthy pregnancies (421.28 ng/mL vs 428.34 ng/mL).

**Conclusion:**

Further studies are needed to establish whether or not sVAP-1 might be considered as an early, non-invasive, and affordable biomarker to screen women who will develop PIH or GDM. Our data will aid sample size calculations for such larger studies.

## Introduction

Pre-eclampsia (PE) is a complex disease affecting pregnant women and a major cause of maternal and perinatal morbidity and mortality worldwide [1]. Its incidence is approximately 5–8%, and each year around 76,000 mothers and 500,000 babies die due to hypertensive disorders of pregnancy (HDP) [2, 3]. Given the great variability of its symptoms and clinical presentation, diagnosis of pre-eclampsia remains a challenge, and is often diagnosed too late, limiting the opportunity for meaningful intervention other than delivery of the baby and the placenta. Currently, screening to predict pre-eclampsia is limited to obtaining an appropriate medical history, and no biomarker has proven to be effective in the first trimester of pregnancy (when intervention is most likely to be effective) [4]. As shown in our recent systematic review with meta-analysis focused on all blood biomarkers used to detect PE, even if Placental Growth Factor (PlGF) and soluble Flt-1 (sFlt-1) are the most widely used biomarkers in clinical practice, their use is limited to later stages of pregnancy [5]. Thus, a robust diagnosis of PE remains a challenge for current clinical practice, and its identification in the first trimester an ambitious goal [6].

Fetal growth restriction (FGR) is a common pregnancy complication affecting 3–7% of gestations [7]. According to the Royal College of Obstetricians and Gynaecologists (RCOG), it is defined as fetal birth weight below the 10^th^ centile [8]. The diagnosis is made via ultrasonographic assessment. It is currently classified according to severity as moderate or severe, according to body parameters into symmetrical or asymmetrical, and according to the onset into early or late-onset FGR [7, 9]. Early-onset FGR and PE are often (39–43%) associated because of a shared pathophysiology of shallow placentation [9, 10].

Gestational diabetes mellitus (GDM) is defined as any degree of glucose intolerance diagnosed during pregnancy, characterised by a hyperglycaemic state. GDM affects around 5% of pregnant women, and women with GDM have a higher risk of developing diabetes mellitus type 2 (DMT2) later in life [11, 12]. The current screening to detect GDM in the UK consists of performing an Oral Glucose Tolerance Test (OGTT) between 24 and 28 gestational weeks (GWs), following which a diagnosis is made if serum glucose levels are higher than 5.6 mmol/L at fasting (OGTT test 1) or higher than 7.8 mmol/L after 2 hours of 75g glucose intake (OGTT test 2) [13, 14].

VAP-1 is an enzyme that is well known for its involvement in promoting leukocyte transmigration towards sites of inflammation [15]. VAP-1 as a biomarker has been studied in several pathological conditions, and VAP-1 has been proposed as a potential indicator of inflammatory diseases [16]. Additionally, it has been shown to contribute to angiogenesis and it has been suggested that this may provide potential for the development of new drugs [17]. VAP-1 has been shown to be the most abundant protein expressed in perivascular cells surrounding the spiral arteries [18].

Further, with reference to pregnancy, sVAP-1 (the soluble form found in serum), has been found to be elevated in normal pregnancy [19]. VAP-1 has been implicated in the early immunological response of the endometrium towards the implanting embryo [20], and this mechanism could lead poor placentation leading to pregnancy complications such as PE and FGR. Thus, sVAP-1 levels may predict adverse pregnancy outcomes.

In this pilot study we measured sVAP-1 serum levels and correlated these with pregnancy complications, in particular, PIH, in order to perform an accurate power calculation for a definitive study.

## Materials and methods

This was a prospective observational pilot study conducted over a four-month period (between July and October 2021) in the Antenatal Clinic of the Leicester Royal Infirmary (University Hospitals of Leicester NHS Trust), an acute tertiary hospital. The study has been registered on ClinicalTrials.gov (Study Identifier: NCT04891315) after being approved by the University Hospitals of Leicester NHS Trust Research Ethics Committee and Women’s and Children’s Clinical Management Group (REC reference 21/EM/0090). Participants were recruited during their first antenatal visit and routine blood tests (before 20 weeks of gestation), and written consent was provided after a thorough and individual explanation of the study details.

All pregnant women attending LRI antenatal clinic in their early pregnancy (<20 gestational weeks), older than 16 years, and willing to participate were considered as being eligible to take part to the study. Women were excluded where they were not willing to participate or if venepuncture was unsuccessfully performed. Written consent was not taken from vulnerable groups, or from women unable to communicate in English when a hospital translator was not immediately available.

### Data collection

Booking data, blood tests performed at booking and at 28 gestational weeks (GWs), fetal anomaly screening data, additional blood tests performed, past pregnancies information, current deliveries data, and maternity assessment unit admission records were all retrospectively collected from hospital records.

### Blood collection and sVAP-1 measurement (ELISA)

Around 5 ml of additional whole blood was withdrawn from each patient by venepuncture during collection of routine booking bloods into Becton Dickinson (BD) ‘brown top’ tubes, containing silica as additive. Following collection, whole blood was centrifuged (≤1300 g for 10 minutes at 18–25° C), aliquoted serum moved to 2.0 ml microfuge tubes and the resulting samples stored at -80°C for future testing. Soluble VAP-1 concentration in the serum was measured using an Enzyme linked immunosorbent assay (ELISA) using the R&D Solid Phase Sandwich ELISA Human VAP-1 Quantikine^®^ kit according to the manufacturers’ instructions. The assay has a minimum sensitivity of 0.077 ng/mL. Samples were assayed either as single points or in some cases as duplicates. Where duplicates were assayed, there was a <5% variation in the interpolated concentrations. VAP-1 levels were interpolated from kit standard curves.

### Statistical analysis

Distribution of continuous variables was checked both graphically (through histograms) and formally (using the Shapiro-Wilk test for normality). In line with the test results, the p-value of each variable was assessed: if lower than 0.05, the variable was considered as skewed (and normality ruled out), if higher, normality was confirmed.

Following this, pregnancy complications were grouped into PIH, fetal growth restriction (FGR), gestational diabetes (GDM), other complications (prolonged spontaneous rupture of membranes (SROM), premature rupture of membrane (PROM), macrosomia, static growth, fetal anomaly, Group B Streptococcus infection, polyhydramnios, miscarriage, placenta praevia, trisomy T21, fetal atrial ectopic beats, maternal anxiety, thyroid problems) and analysed individually. For each of these descriptive analyses first (with the aim of comparing data in women with or without each pregnancy complication) and statistical tests by group after were performed. Following the descriptive analyses by pregnancy complication, statistical significance of the results was assessed using the t-test (for continuous normally distributed variables), the Mann-Whitney test (for continuous not normally distributed variables), or the Pearson chi^2^ test (for categorical variables).

Finally, the aforementioned statistical tests were used to compare differences in blood parameters (including sVAP-1) among women with and without a specific pregnancy complication when statistically relevant (statistical tests assessed parameters measured in one patient only were not performed). Tests performed but not included in the analysis because of a small sample size included alpha fetoprotein (AFP), Oestriol, Inhibin-A, bile acids, amylase, alkaline phosphatase (ALP).

Univariable analyses was carried out to assess associations between VAP-1 levels and pregnancy complications (on a continuous scale). To assess binary outcomes (e.g., those who developed hypertension and those who did not) the Mann-Whitney-U test was used throughout the analysis. To assess associations between VAP-1 and continuous parameters (e.g., age and BMI) univariable regression analyses was used in order to fulfil the secondary study objectives.

Adjusted models were also fitted to assess if other pregnancy complications are associated with VAP-1.

Descriptive analyses (means, medians, and percentages) were used to summarise the whole cohort of women who consented to have a blood sample taken. Descriptive analyses were also used to compare women who developed a pregnancy complication to those who did not.

All statistical analyses were carried out in the software packages Stata 17.0 / April 20, 2021, College Station, TX 77845 USA and Microsoft Excel 2016, Microsoft Corporation, Redmond, WA 98052–7329 USA. The former software was used for creating statistical codes and run the analyses, the latter for collecting and organising data into tables.

### Study power calculation and sample size

The primary outcome of the study was to assess if sVAP-1 levels differ between healthy pregnancies and pregnancies affected by hypertension (which normally comprise of 8–10% of the total) [21]. The only study in literature on sVAP-1 levels during pregnancy reported sVAP-1 levels to be as mean (SD) 2.2 ng/mL (0.74) in healthy pregnancies and as 3.35 ng/mL (1.52) in women with GDM [22]. With a difference of 0.8 ng/mL considered to be clinically significant for 90% power at the 5% significance level, 451 samples were initially considered as needed (roughly 41 with hypertension and 410 normal pregnancies), to detect a difference of mean (SD) 2.2 (1.5) in healthy pregnancies and 3.0 (1.52) in pregnancies with hypertension. Using the data generated from 91 patients a second power calculation was performed and the new results showed that in order to achieve a power of 80% and a significance of 0.05, a total of 9420 recruited participants would have been needed. A decision was taken to terminate the study in favour of a repeat multicentre protocol with this increase recruitment.

## Results

**Fig 1:**
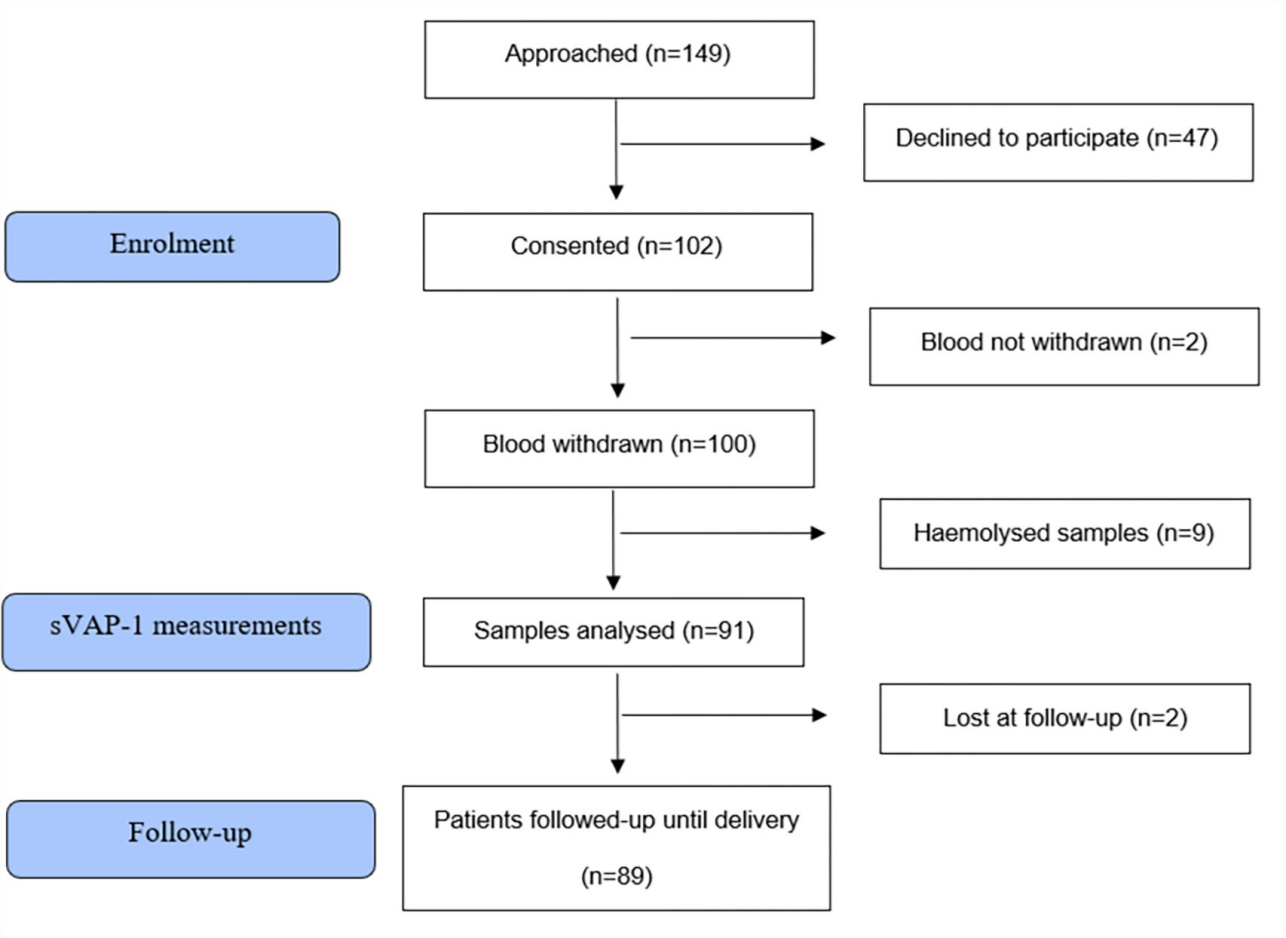
Study flow diagram.

From July to October 2021, 102 patients consented to take part in the study. Following missed blood collection (2 cases) and 9 haemolysed samples, 91 of the enrolled participants had their blood analysed, and 89 were followed up until delivery (Fig 1).

Data obtained from these 91 women were extracted (Table 1). All patients included in these studies had a singleton pregnancy. Overall, the study population appeared to show a variety of ethnic diversity, since 28.08% of the whole sample did not speak English as first language. The other first languages other than English (N = 70) were Romanian (N = 4), Polish (N = 3), Gujarati (N = 3), Malayalam (N = 2), Kurdish (N = 2), Portuguese (N = 1), Italian (N = 1), Greek (N = 1), Slovakian (N = 1) Arabic (N = 1), Igbo (N = 1), Urdu (N = 1). With a median BMI of 26.62 kg/m^2^ (interquartile range 21.88–29.02), the general weight was healthy, and most of the women were secundipara (32.95%). Interestingly, only 53.85% of pregnancies were planned (Table 1). Overall, 2 women were lost at follow up, therefore data regarding their later stages of pregnancy are missing. Table 1 also reports VAP-1 measurements.

**Table 1:** Descriptive analysis for the whole population.

Variable, unit of measurement (N)	Mean (SD)–Median (IQR)–N (%)	p-values from the Shapiro-Wilks test (continuous variables)
Age, years (N = 91)	26.66 (SD 5.15)	0.584
BMI, kg/m^2^ (N = 89)	26.62 (IQR 21.88–29.02)	<0.001
Blood group (N = 90)		
0 Rh+	30 (33.3%)	NA
0 Rh-	3 (3.33%)	
A Rh+	33 (36.67%)	
A Rh-	6 (6.67%)	
B Rh +	13 (14.44%)	
B Rh –	3 (3.33%)	
AB Rh +	1 (1.11%)	
AB Rh -	1 (1.11%)	
Blood Rhesus (N = 90)		
Positive (+)	77 (85.56%)	NA
Negative (-)	13 (14.44%)	
Gravidity (N = 88)		
G1	23 (26.14%)	NA
G2	29 (32.95%)	
G3	10 (11.36%)	
G4	6 (6.82%)	
G5	11 (12.50%)	
G6	2 (2.27%)	
G7	2 (2.27%)	
G8	2 (2.27%)	
G9	2 (2.27%)	
G10	1 (1.14%)	
Parity (N = 88)		
P0	31 (35.23%)	NA
P1	29 (32.95%)	
P2	13 (14.77%)	
P3	6 (6.82%)	
P4	5 (5.68%)	
P5	4 (4.55%)	
Mother Ethnicity (N = 91)		
White	71 (78.02%)	NA
Asian	14 (15.38%)	
Black	5 (5.49%)	
Others	1 (1.10%)	
Baby’s father ethnicity (N = 91)		
White	61 (67.02%)	NA
Asian	19 (20.88%)	
Black	7 (7.69%)	
Others/Unknown	4 (4.44%)	
Smoking (N = 91)		
No-smokers	54 (59.34%)	NA
Smokers	16 (17.58%)	
E-cigarettes users	4 (4.4%)	
Ex-smokers	17 (18.68%)	
Smokers in household (N = 91)		
No	68 (74.73%)	NA
Yes	23 (25.27%)	
Alcohol/Substances abuse (N = 91)		
No	57 (62.64%)	NA
Stopped alcohol	29 (31.87%)	
Stopped drugs	1 (1.10%)	
Current users	4 (4.40%)	
Thromboembolic Risk (N = 91)		
Low	68 (74.73)	NA
Intermediate	8 (8.79%)	
High	15 (16.48%)	
Pregnancy Category of Risk (N = 91)		
Low	43 (47.25%)	NA
High	48 (52.75%)	
Fertility Treatment (N = 91)		
No	88 (96.70%)	NA
Yes	3 (3.30%)	
Planned Pregnancy (N = 91)		
No	37 (40.66%)	NA
Yes	49 (53.85%)	
No (on pill during at conception)	5 (5.49%)	
Outcome of past pregnancies (N = 87)		
Never delivered (P0 or TOP)	25 (28.74%)	NA
Livebirths (P1+)	34 (39.08%)	
Miscarriages, stillbirths, molar pregnancies	28 (32.18%)	
Number of MAU admissions (N = 89)		
0	39 (43.8%)	NA
1	21 (23.6%)	
2	12 (13.48%)	
3	4 (4.49%)	
4	4 (4.49%)	
5	4 (4.49%)	
6	2 (2.25%)	
7	2 (2.25%)	
9	1 (1.12%)	
GW at delivery (N = 88)		
Term (37–42 GW)	79 (89.77%)	NA
Pre-term (</ = 37 GW)	8 (9.09%)	
Post-term (>42 GW)	1 (1.14%)	
EBL, mL (N = 88)	307.5 (IQR 200–500)	<0.001
Infant weight, g (N = 87)	3380 (IQR 3000–3740)	<0.001
Infant Sex (N = 86)		
Females	32 (37.21%)	NA
Males	54 (62.79%)	
Infant Outcome (N = 88)		
Livebirths	86 (97.73%)	NA
Miscarriages or TOPs	2 (2.27)	
sVAP-1, ng/mL (N = N = 91)	427 (IQR 334.28–552.18)	0.033
NT, mm (N = 83)	1.01 (IQR 0.87–1.22)	<0.001
Free beta hCG, IU/L (N = 89)	0.99 (IQR 0.67–1.59)	<0.001
AFP, kU/mL (N = 9)	0.90 (SD 0.20)	0.821
uOestriol, pg/mL (N = 9)	0.99 (SD 0.16)	0.823
Inhibin-A, pg/mL (N = 9)	0.98 (IQR 0.86–1.03)	0.007
PAPP-A, MoM (N = 82)	1.12 (IQR 0.69–1.54)	<0.001
BOOKING BLOODS		
Hb, g/L (N = 91)	129.56 (SD 10.85)	0.774
WCC, 10^9^/L (N = 91)	9.20 (SD 2.33)	0.974
RBC, 10^12^/L (N = 91)	4.39 (SD 0.38)	0.148
Hct, L/L (N = 91)	0.399 (SD 0.03)	0.087
MCV, fL (N = 90)	92 (IQR 88–95)	0.009
Platelets, 10^9^/L (N = 91)	241.66 (SD 53.25)	0.604
Neutrophils, 10^9^/L (N = 91)	6.57 (SD 2.04)	0.838
Eosinophils, 10^9^/L (N = 91)	0.11 (IQR 0.07–0.21)	<0.001
Basophils, 10^9^/L (N = 90)	0.04 (SD 0.02)	0.09997
Monocytes, 10^9^/L (N = 91)	0.47 (IQR 0.4–0.57)	<0.001
Total lymphocytes, 10^9^/L (N = 91)	1.89 (IQR 1.58–2.23)	0.001
28 GW BLOOD TESTS		
OGTT test 1, mmol/L (N = 49)	4.3 (IQR 4.1–4.6)	0.001
OGTT test 2, mmol/L (N = 49)	5.9 (IQR 4.9–7.1)	0.0098
Hb, g/L (N = 88)	117.38 (SD 9.97)	0.593
WCC, 10^9^/L (N = 88)	10.4 (IQR 8.25–12.25)	0.039
RBC, 10^12^/L (N = 88)	3.95 (SD 0.39)	0.999
Hct, L/L (N = 88)	0.351 (SD 0.03)	0.617
MCV, fL (N = 88)	89.10 (SD 6.52)	0.933
Platelets, 10^9^/L (N = 88)	266.43 (SD 64.20)	0.373
Neutrophils, 10^9^/L (N = 88)	7.71 (IQR 6.21–9.23)	0.007
Eosinophils, 10^9^/L (N = 88)	0.12 (IQR 0.08–0.18)	<0.001
Basophils, 10^9^/L (N = 88)	0.03 (IQR 0.02–0.04)	0.001
Monocytes, 10^9^/L (N = 88)	0.49 (IQR 0.42–0.59)	<0.001
Total Lymphocytes, 10^9^/L (N = 88)	1.84 (IQR 1.61–2.4)	<0.001
OTHER BLOOD TESTS		
ALT, U/L (N = 20)	15 (IQR 14–17)	<0.001
Albumin, g/L N = (23)	41.74 (SD 3.02)	0.781
ALP, U/L (N = 22)	87.73 (SD 31.67)	0.688
Total bilirubin, μmol/L (N = 23)	6 (IQR 4–7)	<0.001
Total protein, g/L (N = 23)	67 (IQR 64–70)	0.046
Amylase, U/L (N = 6)	74.5 (SD 20.40)	0.914
Creatinine, μmol/L (N = 31)	44 (IQR 40–49)	<0.001
Potassium, mmol/L (N = 32)	4.07 (SD 0.36)	0.214
Sodium, mmol/L (N = 32)	137 (IQR 135.5–138)	<0.001
Urea, mmol/L (N = 32)	2.9 (SD 0.57)	0.994
HbA1c, % (N = 24)	5.2 (IQR 5–5.5)	0.0455
HbA1c, mmol/mol (N = 24)	34.75 (SD 4.86)	0.053
Protein/creatinine ratio, mg/mmol (N = 18)	13.8 (IQR 11.6–16.9)	<0.001
Random protein, mg/dl (N = 19)	0.2 (IQR 0.19–0.3)	0.002
Random creatinine, mg/dL (N = 23)	15.09 (SD 7.80)	0.496
APTT, s (N = 10)	28.06 (SD 1.50)	0.674
APTT ratio (N = 10)	1 (IQR 1–1)	0.002
INR (N = 10)	0.95 (SD 0.05)	0.99999
Prothrombin time, s (N = 10)	12.92 (SD 0.67)	0.853
Vitamin D, nmol/L (N = 2)	42 (SD 21.21)	NA
Serum folate, mg/mL (N = 5)	11.6 (SD 4.74)	0.151
Serum ferritin, μg/L (N = 7)	16.57 (SD 11.91)	0.096
Serum Vitamin B12, ng/L (N = 4)	382.75 (SD 92.08)	0.970
TSH, mU/L (N = 9)	1.14 (SD 0.80)	0.941
Free thyroxine, pmol/L (N = 5)	14.8 (SD 2.28)	0.360
Free T3, pmol/L (N = 2)	4.6 (SD 0.28)	NA

Normally distributed variables are described in terms of their mean value and standard deviation (SD). Skewed variables are summarised using the respective median (50%) and interquartile range (IQR); standard deviations and interquartile ranges (25%-75%) are reported as measures of spread. Categorical variables are described in terms of numbers (N) and percentages (%).

As shown in Table 1, 100% and 80.08% of the recruited women had a full blood count performed at booking and at 28 GW, respectively. As expected, following physiological changes occurring during pregnancy, haemoglobin, red blood cell (RBC) count, and haematocrit levels were higher overall in early pregnancy (average difference of 12.48, 0.45, 0.05, respectively). White Cell Counts (WCCs) are also increased in the late second trimester.

### Descriptive analyses by pregnancy complication

The first descriptive analysis was performed for all the variables, and each one was assessed by pregnancy induced hypertension (PIH). Consequently, the study population was divided into women with or without PIH and, as shown in Table 2, only seven gestations developed hypertension. Overall, the hypertensive population appeared to be younger (27.57 vs 29.72 years) and with a slightly higher BMI (27.55 vs 24.62 kg/m^2^) than the control one. Interestingly, no woman with PIH had a blood group B or AB nor a Rhesus negative blood type: all PIH women were either 0 Rh+ or A Rh +. Most of them (57.14%) were nullipara. All the women (100%) who developed PIH were white; the ethnicity of the father was white in all cases. In line with the claimed role of cigarettes use in decreasing the risk of developing PIH [23], 57.14% of the PIH study population was an ex-smoker. Interestingly, infant weight was lower in babies born from PIH women as compared to the control group (3000 vs 3390 g), reinforcing the well-established association between PIH and FGR [24].

**Table 2:** Descriptive analysis and statistical tests by PIH.

VARIABLE (N)	PIH (N = 7): Mean (SD)–Median (IQR)–N(%)	no PIH (N = 81): Mean (SD)–Median (IQR)–N(%)	p-values
AGE (N = 81)	27.57 (SD 3.87)	29.72 (SD 5.25)	0.295
BMI (N = 89)	27.55 (IQR 26.08–33.96)	24.62 (IQR 21.73–29.39)	0.095
BLOOD TYPE (N = 90)			0.536
0	2 (33.33)	31 (38.27)	
A	4 (66.67)	33 (40.74)	
B	0 (0)	15 (18.52)	
AB	0 (0)	2 (2.47)	
**BLOOD RHESUS (N = 90)**			0.287
** Rh +**	6 (100)	68 (83.95)	
** Rh -**	0 (0)	13 (16.05)	
**GRAVIDITY (N = 88)**			0.586
** G1**	3 (42.86)	19 (24.36)	
** G2**	2 (28.57)	26 (33.33)	
** G3**	0 (0)	10 (12.82)	
** G4**	0 (0)	5 (6.41)	
** G5**	1 (14.29)	10 (12.82)	
** G6**	0 (0)	2 (2.56)	
** G7**	0 (0)	2 (2.56)	
** G8**	1 (14.29)	1 (1.28)	
** G9**	0 (0)	2 (2.56)	
** G10**	0 (0)	1 (1.28)	
**PARITY (N = 88)**			0.486
** P0**	4 (57.14)	25 (32.05)	
** P1**	1 (14.29)	28 (35.90)	
** P2**	1 (14.29)	12 (15.38)	
** P4**	0 (0)	5 (6.41)	
** P4**	0 (0)	5 (6.41)	
** P5**	1 (14.29)	3 (3.85)	
**MOTHER ETHNICITY (N = 91)**			0.553
** White**	7 (100)	62 (76.54)	
** Asian**	0 (0)	13 (16.05)	
** Black**	0 (0)	5 (6.17)	
** Others**	0 (0)	1 (1.23)	
**BABY’S FATHER ETHNICITY (N = 91)**			0.291
** White**	7 (100)	52 (64.20)	
** Asian**	0 (0)	18 (22.22)	
** Black**	0 (0)	7 (8.64)	
** Others**	0 (0)	4 (4.94)	
**SMOKING (N = 91)**			0.015
** No**	2 (28.57)	50 (61.73)	
** Yes (cigarettes)**	0 (0)	16 (19.75)	
** Yes (e-cigarette)**	1 (14.29)	3 (3.70)	
** Yes, but stopped**	4 (57.14)	12 (14.8)	
**SMOKERS IN HOUSEHOLD (N = 91)**			0.041
** No**	3 (42.86)	63 (77.78)	
** Yes**	4 (57.14)	18 (22.22)	
**ALCOHOL/SUBSTANCE ABUSE (N = 91)**			0.859
** No**	4 (57.14)	51 (62.96)	
** Stopped alcohol**	3 (42.86)	25 (30.86)	
** Stopped drugs**	0 (0)	1 (1.23)	
** Yes**	0 (0)	4 (4.94)	
**THROMBOEMBOLIC RISK (N = 91)**			0.139
** Low**	4 (57.14)	61 (75.31)	
** Intermediate**	0 (0.0)	8 (9.88)	
** High**	3 (42.86)	12 (14.81)	
**PREGNANCY CATEGORY OF RISK (N = 91)**			0.350
** Low**	2 (28.57)	38 (46.91)	
** High**	5 (71.43)	43 (53.09)	
**FERTILITY TREATMENT (N = 91)**			0.604
** No**	7 (100)	78 (96.3)	
** Yes**	0 (0)	3 (3.70)	
**PLANNED PREGNANCY (N = 91)**			0.017
** No**	1 (14.29)	33 (40.74)	
** Yes**	4 (57.14)	45 (55.56)	
** No (on pill during conception)**	2 (28.57)	3 (3.70)	
**OUTCOME OF PAST PREGNANCIES (N = 87)**			0.671
** Never delivered (P0 or TOP)**	3 (42.86)	21 (27.27)	
** Livebirths (P1+)**	2 (28.57)	31 (40.26)	
** Miscarriages, stillbirths, molar pregnancies**	2 (28.57)	25 (32.47)	
**NUMBER OF MAU ADMISSIONS (N = 89)**			<0.001
** 0**	0 (0)	38 (46.91)	
** 1**	3 (42.86)	18 (22.22)	
** 2**	0 (0)	12 (14.81)	
** 3**	0 (0)	4 (4.94)	
** 4**	1 (14.29)	3 (3.70)	
** 5**	2 (28.57)	2 (2.47)	
** 6**	0 (0)	2 (2.47)	
** 7**	0 (0)	2 (2.47)	
** 8**	1 (14.29)	0 (0)	
**GW DELIVERY (N = 88)**			0.850
** Term**	6 (85.71)	73 (90.12)	
** Pre-term**	1 (14.29)	7 (8.64)	
** Post-term**	0 (0)	1 (1.23)	
**EBL (N = 88)**	350 (IQR 200–600)	300 (IQR 200–500)	0.919
**INFANT WEIGHT (N = 87)**	3000 (IQR 2600–4060)	3390 (IQR 3045–3740)	0.617
**INFANT SEX (N = 86)**			0.622
** Female**	2 (28.57)	30 (37.97)	
** Male**	5 (71.43)	49 (62.03)	
**INFANT OUTCOME (N = 88)**			0.674
** Livebirth**	7 (100)	79 (97.53)	
** Miscarriage / TOP**	0 (0)	2 (2.47)	

The same descriptive analysis was performed for the study population by FGR, revealing data similar to PIH in terms of age, BMI, and blood Rhesus (Table 3). A total of 7 babies were born small for their gestational age. As expected, a difference between FGR fetuses’ weight and the weight of infants with a normal growth pattern was found (2540 g vs 3470 g, respectively). As for PIH, FGR appears to affect males more commonly than females (Tables 2 and 3).

**Table 3:** Descriptive analysis and statistical tests by FGR.

VARIABLES (N)	FGR (N = 7): Mean (SD)–Median (IQR)–N(%)	no FGR (N = 81): Mean (SD)–Median (IQR)–N(%)	p-values
AGE (N = 81)	26.86 (SD 3.44)	29.78 (SD 5.24)	0.153
BMI (N = 89)	30.74 (IQR 20.9–31.63)	24.67 (IQR 22.01–28.67)	0.522
BLOOD TYPE (N = 90)			
0	2 (33.33)	31 (38.27)	0.966
A	3 (50)	34 (41.98)	
B	1 (16.67)	14 (17.28)	
AB	0 (0)	2 (2.47)	
BLOOD RHESUS (N = 90)			
Rh +	6 (100)	68 (83.5)	0.287
Rh -	0 (0)	13 (16.05)	
GRAVIDITY (N = 88)			
G1	0 (0)	22 (28.21)	0.403
G2	3 (42.86)	25 (32.05)	
G3	1 (14.29)	9 (11.54)	
G4	0 (0)	5 (6.41)	
G5	2 (28.57)	9 (11.54)	
G6	0 (0)	2 (2.56)	
G7	0 (0)	2 (2.56)	
G8	1 (14.29)	1 (1.28)	
G9	0 (0)	2 (2.56)	
G10	0 (0)	1 (1.28)	
PARITY (N = 88)			
P0	1 (14.29)	28 (35.90)	0.505
P1	3 (42.86)	26 (33.33)	
P2	2 (28.57)	11 (14.10)	
P3	0 (0)	5 (6.41)	
P4	0 (0)	5 (6.41)	
P5	1 (14.29)	3 (3.85)	
MOTHER ETHNICITY (N = 91)			
White	6 (85.71)	63 (77.78)	0.903
Asian	1 (14.29)	12 (14.81)	
Black	0 (0)	5 (6.17)	
Others	0 (0)	1 (1.23)	
BABY’S FATHER ETHNICITY (N = 91)			
White	6 (85.71)	53 (65.43)	0.685
Asian	1 (14.29)	17 (20.99)	
Black	0 (0)	7 (8.64)	
Others	0 (0)	4 (4.94)	
SMOKING (N = 91)			
No	5 (71.43)	47 (58.02)	0.498
Yes (cigarettes)	2 (28.57)	14 (17.28)	
Yes (e-cigarette)	0 (0)	4 (4.94)	
Yes, but stopped	0 (0)	16 (19.75)	
SMOKERS IN HOUSEHOLD (N = 91)			
No	5 (71.43)	61 (75.31)	0.820
Yes	2 (28.57)	20 (24.69)	
ALCOHOL/SUBSTANCES ABUSE (N = 91)			
No	5 (71.43)	50 (61.73)	0.907
Stopped alcohol	2 (28.57)	26 (32.10)	
Stopped drugs	0 (0)	1 (1.23)	
Yes	0 (0)	4 (4.94)	
THROMBOEMBOLIC RISK (N = 91)			
Low	4 (57.14)	61 (75.31)	0.174
Intermediate	2 (28.57)	6 (7.41)	
High	1 (14.29)	14 (17.28)	
PREGNANCY CATEGORY OF RISK (N = 91)			
Low	2 (28.57)	38 (46.91)	0.350
High	5 (71.43)	43 (53.09)	
FERTILITY TREATMENT (N = 91)			
No	7 (100)	78 (96.30)	0.604
Yes	0 (0)	3 (3.70)	
PLANNED PREGNANCY (N = 91)			
No	1 (14.29)	33 (40.74)	0.017
Yes	4 (57.14)	45 (55.56)	
No (on pill during conception)	2 (28.57)	3 (3.70)	
OUTCOME OF PAST PREGNANCIES (N = 87)			
Never delivered (P0 or TOP)	0 (0)	24 (31.17)	0.048
Livebirths (P1+)	2 (28.57)	31 (40.26)	
Miscarriages, stillbirths, molar pregnancies	5 (71.43)	22 (28.57)	
NUMBER OF MAU ADMISSIONS (N = 89)			
0	2 (28.57)	36 (44.44)	0.319
1	1 (14.29)	20 (24.69)	
2	2 (28.57)	10 (12.35)	
3	0 (0)	4 (4.94)	
4	0 (0)	4 (4.94)	
5	1 (14.29)	3 (3.70)	
6	0 (0)	2 (2.47)	
7	1 (14.29)	1 (1.23)	
8	0 (0)	1 (1.23)	
GW DELIVERY (N = 88)			
Term	4 (57.14)	75 (92.59)	0.005
Pre-term	3 (42.86)	5 (6.17)	
Post-term	0 (0)	1 (1.23)	
EBL (N = 88)	250 (IQR 200–400)	350 (IQR 200–500)	0.335
INFANT WEIGHT (N = 87)	2540 (IQR 1700–3270)	3470 (IQR 3055–3745)	0.008
INFANT SEX (N = 86)			
Female	2 (28.57)	30 (37.97)	0.622
Male	5 (71.43)	49 (62.03)	
INFANT OUTCOME (N = 88)			
Livebirth	7 (100)	79 (97.53)	0.674
Miscarriage / TOP	0 (0)	2 (2.47)	

GDM was the last pregnancy complication individually assessed using descriptive analysis. Overall, 10 out of 81 women developed GDM during the pregnancy object of our study: among them, a slightly older age and higher BMI must be noted (Table 4). Equally to the previously assessed pregnancy complications, 100% of women with GDM was Rh +. 40% of GDM women had an Asian ethnicity, and 40% were white; up to 50% of male partners were Asian. Smoking did not appear to be a predisposing factor, since none of the women who developed GDM was an active smoker.

**Table 4:** Descriptive analysis and statistical tests by GDM.

VARIABLES (N)	GDM (N = 10): Mean (SD)–Median (IQR)–N(%)	no GDM (N = 78): Mean (SD)–Median (IQR)–N(%)	p-values
AGE (N = 81)	29.7 (SD 3.89)	29.53 (SD 5.33)	0.921
BMI (N = 89)	28.85 (IQR 24.56–32.87)	24.62 (IQR 21.67–28.66)	0.027
BLOOD TYPE (N = 90)			
0	4 (40)	29 (37.66)	0.624
A	3 (30)	34 (44.16)	
B	3 (30)	12 (15.58)	
AB	0 (0)	2 (2.60)	
BLOOD RHESUS (N = 90)			
Rh +	10 (100)	64 (83.12)	0.159
Rh -	0 (0)	13 (16.88)	
GRAVIDITY (N = 88)			
G1	2 (20)	20 (26.67)	0.334
G2	1 (10)	27 (36)	
G3	2 (20)	8 (10.67)	
G4	2 (20)	3 (4)	
G5	3 (30)	8 (10.67)	
G6	0 (0)	2 (2.67)	
G7	0 (0)	2 (2.67)	
G8	0 (0)	2 (2.67)	
G9	0 (0)	2 (2.67)	
G10	0 (0)	1 (1.33)	
PARITY (N = 88)			
P0	3 (30)	26 (34.67)	0.029
P1	1 (10)	28 (37.33)	
P2	5 (50)	8 (10.67)	
P3	0 (0)	5 (6.67)	
P4	1 (10)	4 (5.33)	
P5	0 (0)	4 (5.33)	
MOTHER ETHNICITY (N = 91)			
White	4 (40)	65 (83.33)	0.002
Asian	4 (40)	9 (11.54)	
Black	1 (10)	4 (5.13)	
Others	1 (10)	0 (0)	
BABY’S FATHER ETHNICITY (N = 91)			
White	3 (30)	56 (71.79)	0.048
Asian	5 (50)	13 (16.67)	
Black	1 (10)	6 (7.69)	
Others	1 (10)	3 (3.85)	
SMOKING (N = 91)			
No	8 (80)	44 (56.41)	0.331
Yes (cigarettes)	0 (0)	16 (20.51)	
Yes (e-cigarette)	0 (0)	4 (5.13)	
Yes, but stopped	2 (20)	14 (17.95)	
SMOKERS IN HOUSEHOLD (N = 91)			
No	8 (80)	58 (74.36)	0.698
Yes	2 (20)	20 (25.64)	
ALCOHOL/SUBSTANCES ABUSE (N = 91)			
No	8 (80)	47 (60.26)	0.639
Stopped alcohol	2 (20)	26 (33.33)	
Stopped drugs	0 (0)	1 (1.28)	
Yes	0 (0)	4 (5.13)	
THROMBOEMBOLIC RISK (N = 91)			
Low	7 (70)	58 (73.36)	0.340
Intermediate	0 (0)	8 (10.26)	
High	3 (30)	12 (15.38)	
PREGNANCY CATEGORY OF RISK (N = 91)			
Low	1 (10)	39 (50)	0.017
High	9 (90)	39 (50)	
FERTILITY TREATMENT (N = 91)	No = 10 (100)	No = 75 (96.15)	0.528
	Yes = 0 (0)	Yes = 3 (3.85)	
PLANNED PREGNANCY (N = 91)			
No	4 (40)	30 (38.4)	0.710
Yes	6 (60)	43 (55.13)	
No (on pill during conception)	0 (0)	5 (6.41)	
OUTCOME OF PAST PREGNANCIES (N = 87)			
Never delivered (P0 or TOP)	2 (20)	22 (29.73)	0.434
Livebirths (P1+)	3 (30)	30 (40.54)	
Miscarriages, stillbirths, molar	5 (50)	22 (29.73)	
pregnancies			
NUMBER OF MAU ADMISSIONS (N = 89)			
0	3 (30)	35 (44.87)	0.891
1	3 (30)	18 (23.08)	
2	2 (20)	10 (12.82)	
3	1 (10)	3 (3.85)	
4	1 (10)	3 (3.85)	
5	0 (0)	4 (5.13)	
6	0 (0)	2 (2.56)	
7	0 (0)	2 (2.56)	
8	0 (0)	1 (1.28)	
GW DELIVERY (N = 88)			
Term	9 (90%)	70 (89.74%)	0.933
Pre-term	1 (10%)	7 (8.78%)	
Post-term	0 (0%)	1 (1.28%)	
EBL (N = 88)	250 (IQR 150–350)	350 (IQR 200–500)	0.086
INFANT WEIGHT (N = 87)	3155 (IQR 2700–3600)	3400 (IQR 3040–3750)	0.223
INFANT SEX (N = 86)			
Female	2 (20)	30 (39.47)	0.231
Male	8 (80)	46 (60.53)	
INFANT OUTCOME (N = 88)			
Livebirth	10 (100)	76 (97.44)	0.608
Miscarriage / TOP	0 (0)	2 (2.56)	

Any pregnancy complication other than PIH, FGR, and GDM was grouped and analysed separately, but no relevant difference in terms of age nor BMI can be highlighted between the disease and the control groups (S1 Table). A total of 16 women experienced at least one of the following: prolonged spontaneous rupture of membranes (SROM), premature rupture of membranes (PROM), macrosomia, static growth, fetal anomalies, Group B Streptococcus infection, polyhydramnios, miscarriage, placenta praevia, trisomy 21, fetal atrial ectopic beats, maternal anxiety, thyroid issues.

Finally, patients with any of the already analysed pregnancy complications were comprehensively compared to women with a healthy gestation (S2 Table). Overall, no statistically significant difference was found between the two groups. Among the ones who experienced complications, 40% experienced an adverse pregnancy outcome (miscarriage, molar pregnancy, stillbirth) in their past maternal history, pointing out at a plausible recurrence of pregnancy complications in the same woman throughout different gestations [25]. The median birthweight was generally lower among those patients (3090 g) as compared to healthy controls (3500 g).

### sVAP-1 measurements

#### Statistical correlations between pregnancy complications

**Fig 2:**
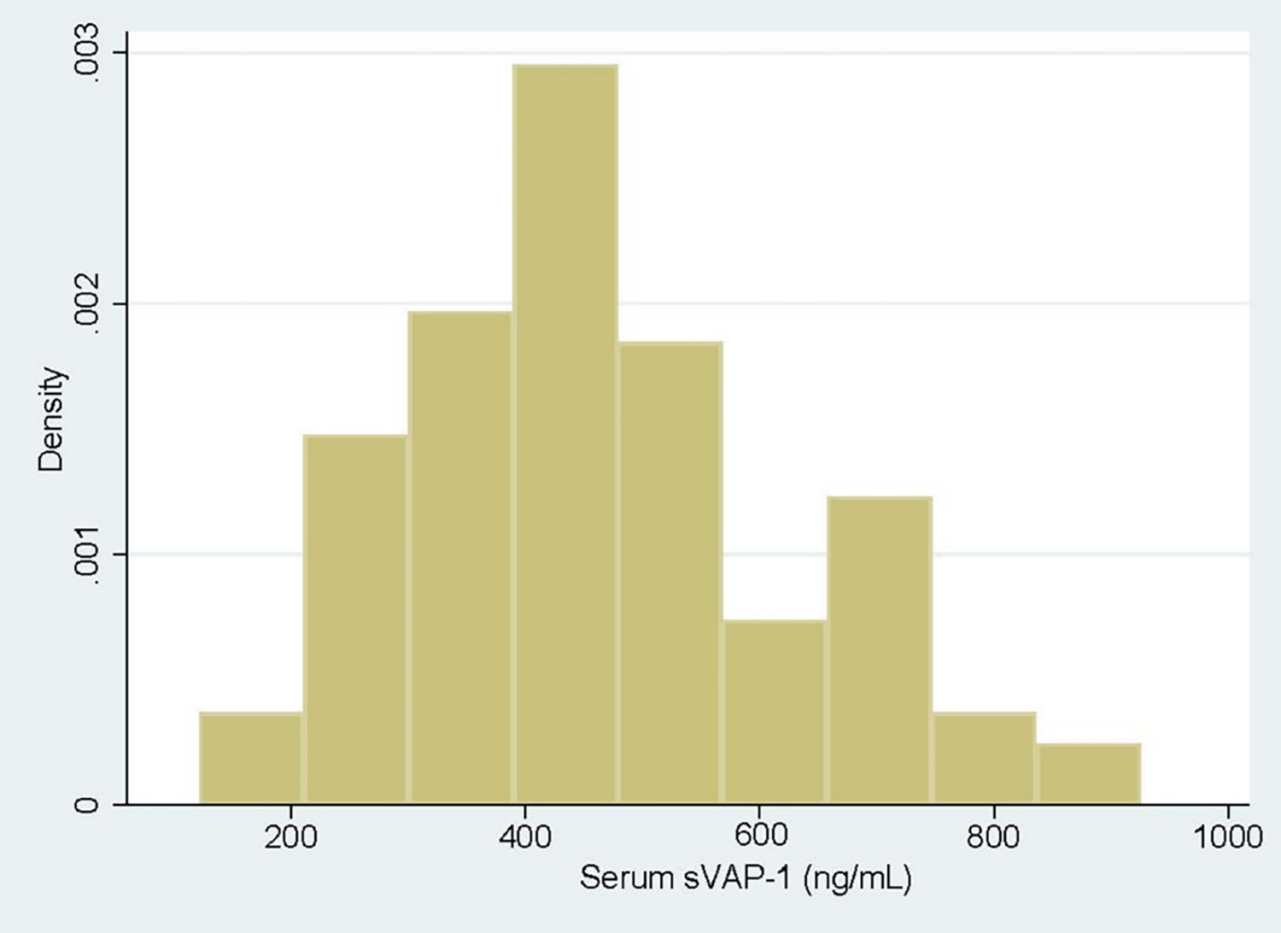
Histogram of sVAP-1 serum concentrations.

Soluble VAP-1 concentrations were all obtained between the late first and the second trimester and vary from 121.64 to 924.4 ng/mL, with a median value of 427.44 ng/mL (IQR 334.28–552.18). VAP-1 normality was assessed using Shapiro-Wilk test and classified as continuous not normally distributed (skewed) (Fig 2).

Following the descriptive analyses, statistical correlations between disease biomarkers and outcome of pregnancy were carried out (Tables 5–7). As shown in Table 5 (and in line to what was suggested by some preliminary data obtained before starting this study), the median value of sVAP-1 in the PIH population was lower than compared to the normotensive population (310.33 ng/mL versus 427.44 ng/mL). Consequently, our findings support the initial hypothesis of sVAP-1 as being a potential early biomarker of hypertension during pregnancy. Women affected by PIH in our study appear to have higher levels of white cell count (WCC) and neutrophils at both booking and 28 GW as compared to non-hypertensive women. Raised haemoglobin, mean cell volume (MCV), red blood cells (RBC), haematocrit (Hct) throughout gestation are associated with hypertension, too. On the other hand, PIH platelets are slightly decreased in number, as well as PAPP-A–an already known biomarker for pre-eclampsia. PIH women showed a worse liver function, with higher levels of alkaline phosphatase (ALP), alanine transaminase (ALT), and bilirubin. Finally, our results show lower free-beta hCG levels in hypertense women (Table 5), and this correlation is in line with some studies [26] but in contrast to others [27].

**Table 5:** Statistical correlations between PIH and blood biomarkers.

BIOMARKER (N)	PIH (N = 7): Mean (SD)–Median (IQR)	No PIH (N = 81): Mean (SD)–Median (IQR)	p-values
sVAP-1 (N = 7, 81)	310.33 (IQR 242.68–433.56)	427.44 (IQR 347.38–552.18)	0.021
NT (N = 6, 74)	0.94 (IQR 0.9–1.21)	1.01 (IQR 0.85–1.22)	0.855
Free beta hCG (N = 7,79)	0.88 (IQR 0.56–1.18)	0.99 (IQR 0.69–1.7)	0.198
PAPP-A (N = 6, 73)	1.10 (IQR 0.74–1.4)	1.13 (IQR 0.73–1.54)	0.818
Hb at booking (N = 7, 81)	139.14 (SD 10.54)	129 (SD 10.48)	0.016
WCC at booking (N = 7, 81)	10.11 (SD 1.88)	9.10 (SD 2.38)	0.275
RBC at booking (N = 7, 81)	4.60 (SD 0.39)	4.38 (SD 0.38)	0.147
Hct at booking (N = 7, 81)	0.43 (SD 0.04)	0.40 (SD 0.03)	0.011
MCV at booking (N = 7, 80)	94 (IQR 92–96)	92 (IQR 87.5–94.5)	0.245
Platelets at booking (N = 7, 81)	223.71 (SD 42.28)	243.407 (SD 52.89)	0.341
Neutrophils at booking (N = 7, 81)	7.32 (SD 1.68)	6.47 (SD 2.08)	0.297
Eosinophils at booking (N = 7, 81)	0.12 (IQR 0.07–0.15)	0.11 (IQR 0.07–0.21)	0.877
Basophils at booking (N = 7, 80)	0.04 (SD 0.02)	0.04 (SD 0.02)	0.765
Monocytes at booking (N = 7, 81)	0.52 (IQR 0.44–0.65)	0.46 (IQR 0.39–0.57)	0.239
Total lymphocytes at booking (N = 7, 81)	1.97 (IQR 1.79–2.38)	1.88 (IQR 1.58–2.23)	0.224
OGTT, test 1 (N = 5, 43)	4.2 (IQR 4.1–4.3)	4.4 (IQR 4.1–4.6)	0.254
OGTT, test 2 (N = 5, 43)	6.5 (IQR 4.6–8.5)	5.9 (IQR 4.9–7.1)	0.712
Hb at 28 GW (N = 7, 80)	128.14 (SD 7.67)	116.51 (SD 9.67)	0.003
WCC at 28 GW (N = 7, 80)	11.3 (IQR 10.4–12.4)	9.9 (IQR 8.1–12.2)	0.119
RBC at 28 GW (N = 7, 80)	4.30 (SD 0.43)	3.92 (SD 0.37)	0.014
Hct at 28 GW (N = 7, 80)	0.38 (SD 0.19)	0.35 (SD 0.29)	0.005
MCV at 28 GW (N = 7, 80)	89.43 (SD 5.59)	89.02 (SD 6.65)	0.877
Platelets at 28 GW (N = 7, 80)	264 (SD 55.28)	267.75 (SD 64.85)	0.883
Neutrophils at 28 GW (N = 7, 80)	8.95 (IQR 7.98–9.93)	7.11 (IQR 5.92–9.23)	0.057
Eosinophils at 28 GW (N = 7, 80)	0.14 (IQR 0.09–0.15)	0.12 (IQR 0.08–0.19)	0.804
Basophils at 28 GW (N = 7, 80)	0.03 (IQR 0.01–0.04)	0.03 (IQR 0.01–0.05)	0.422
Monocytes at 28 GW (N = 7, 80)	0.49 (IQR 0.4–0.6)	0.49 (IQR 0.34–0.59)	0.671
Total lymphocytes at 28 GW (N = 7, 80)	1.76 (IQR 1.53–2.28)	1.85 (IQR (1.61–2.15)	0.775
ALT (N = 4, 16)	18.5 (IQR 14.5–35)	15 (IQR 13–16.5)	0.220
Albumin (N = 4, 19)	40.25 (SD 2.5)	42.06 (SD 3.08)	0.288
ALP (N = 4, 18)	94 (SD 38.51)	86.33 (SD 21.08)	0.672
Total bilirubin (N = 4, 19)	6.5 (IQR 5–7.5)	6 (IQR 4–7)	0.768
Total protein (N = 4, 19)	65 (IQR 63–68.5)	68 (IQR 64–70)	0.425
Creatinine (N = 4, 27)	44.5 (IQR 40–50)	44 (IQR 38–49)	0.898
Potassium (N = 4, 28)	4.25 (SD 0.31)	4.04 (SD 0.36)	0.279
Sodium (N = 4, 28)	137.5 (IQR 136.5–138)	137 (IQR 135–138)	0.512
Urea (N = 4, 28)	2.4 (SD 0.41)	2.97 (SD 0.56)	0.061
HbA1c, % (N = 2, 22)	5.4 (IQR 5.2–5.6)	5.2 (IQR 5–5.4)	0.573
HbA1c (N = 2, 22)	35.5 (SD 3.53)	34.68 (SD 5.02)	0.825
Protein/creatinine ratio (N = 2, 16)	13.8 (IQR 13.6–14)	13.6 (IQR 11.45–19.4)	1.000
Random protein (N = 2, 17)	0.19 (IQR 0.08–0.3)	0.2 (IQR 0.19–0.29)	0.655
Random creatinine (N = 2, 21)	13.65 (SD 10.96)	15.22 (SD 7.79)	0.792

**Table 6:** Statistical correlations between FGR and blood biomarkers.

BIOMARKER (N)	Patients with FGR (N = 7): Mean (SD)–Median (IQR)	Patients without FGR (N = 81): Mean (SD)–Median (IQR)	p-values
sVAP-1 (N = 7, 81)	424.32 (IQR 343.53–694.78)	424.52 (IQR 331.98–519.35)	0.565
NT (N = 6, 74)	1.31(IQR 0.9–1.55)	0.99 (IQR 0.87–1.16)	0.141
Free beta hCG (N = 7, 79)	1.18 (IQR 0.53–1.7)	0.99 (IQR 0.67–1.59)	0.838
PAPP-A (N = 6, 73)	0.94 (IQR 0.61–1.54)	1.12 (IQR 0.76–1.49)	0.483
Hb at booking (N = 7, 81)	126.86 (SD 13.95)	130.06 (SD 10.53)	0.454
WCC at booking (N = 7, 81)	8.11 (SD 2.70)	9.27 (SD 2.32)	0.214
RBC at booking (N = 7, 81)	4.26 (SD 0.33)	4.41 (SD 0.39)	0.333
Hct at booking (N = 7, 81)	0.39 (SD 0.03)	0.40 (SD 0.03)	0.535
MCV at booking (N = 7, 80)	91 (IQR 88–95)	92 (IQR 88–94.5)	0.997
Platelets at booking (N = 7, 81)	220 (SD 68.10)	243.73 (SD 50.69)	0.251
Neutrophils at booking (N = 7, 81)	5.47 (SD 2.06)	6.63 (SD 2.04)	0.151
Eosinophils at booking (N = 7, 81)	0.06 (IQR 0.05–0.08)	0.12 (IQR 0.07–0.21)	0.006
Basophils at booking (N = 7, 80)	0.03 (SD 0.01)	0.04 (SD 0.02)	0.129
Monocytes at booking (N = 7, 81)	0.42 (IQR 0.36–0.53)	0.47 (IQR 0.4–0.58)	0.415
Total lymphocytes at booking (N = 7, 81)	1.89 (IQR 1.43–2.44)	1.89 (IQR 1.6–2.21)	0.889
OGTT, test 1 (N = 3, 45)	4.2 (IQR 4.1–5.5)	4.3 (IQR 4.1–4.6)	0.917
OGTT, test 2 (N = 3, 45)	5.3 (IQR 5.3–10.7)	6.1 (IQR 4.9–7.1)	0.865
Hb at 28 GW (N = 7, 80)	118.57 (SD 14.56)	117.35 (SD 9.63)	0.759
WCC at 28 GW (N = 7, 80)	9.4 (IQR 7.1–11.9)	10.4 (IQR 8.35–12.3)	0.432
RBC at 28 GW (N = 7, 80)	3.83 (SD 0.39)	3.97 (SD 0.39)	0.385
Hct at 28 GW (N = 7, 80)	0.35 (SD 0.03)	0.35 (SD 0.03)	0.738
MCV at 28 GW (N = 7, 80)	92.43 (SD 8.89)	88.76 (SD 6.28)	0.156
Platelets at 28 GW (N = 7, 80)	243.71 (SD 65.47)	269.52 (63.71)	0.308
Neutrophils at 28 GW (N = 7, 80)	6.67 (IQR 5.16–9.6)	7.91 (IQR 6.23–9.23)	0.468
Eosinophils at 28 GW (N = 7, 80)	0.07 (IQR 0.04–0.14)	0.12 (IQR 0.9–0.19)	0.159
Basophils at 28 GW (N = 7, 80)	0.02 (IQR 0.01–0.03)	0.03 (IQR 0.02–0.05)	0.011
Monocytes at 28 GW (N = 7, 80)	0.41 (IQR 0.34–0.46)	0.5 (IQR 0.44–0.59)	0.007
Total lymphocytes at 28 GW (N = 7, 80)	1.94 (IQR 1.14–2.44)	1.82 (IQR 1.62–2.15)	0.846
ALT (N = 3, 17)	15 (IQR 10–15)	15 (IQR 14–17)	0.374
Albumin (N = 3, 20)	42.33 (SD 2.31)	41.65 (SD 3.15)	0.724
ALP (N = 3, 19)	103 (SD 29.21)	85.32 (SD 32.10)	0.382
Total bilirubin (N = 3, 20)	6 (IQR 6–19)	5.5 (IQR 4–7)	0.379
Total protein (N = 3, 20)	66 (IQR 64–72)	67.5 (IQR 63.5–70)	0.990
Creatinine (N = 4, 27)	50.5 (IQR 49.5–51.5)	43 (IQR 38–47)	0.018
Potassium (N = 4, 28)	4.2 (SD 0.41)	4.05 (SD 0.36)	0.432
Sodium (N = 4, 28)	137.5 (IQR 87.5–139)	137 (IQR (135.5–138)	0.615
Urea (N = 4, 28)	2.9 (SD 0.44)	2.9 (SD 0.60)	1.000
HbA1c, % (N = 2, 22)	5.75 (IQR 5.2–6.3)	5.2 (IQR 5–5.4)	0.290
HbA1c (N = 2, 22)	39 (SD 8.49)	34.36 (SD 4.53)	0.203
Protein/creatinine ratio (N = 4, 14)	15.7 (IQR 13.8–24.3)	13 (IQR 11.3–15.3)	0.157
Random protein (N = 4, 15)	0.24 (IQR 0.20–0.31)	0.2 (IQR 0.15–0.3)	0.685
Random creatinine (N = 4, 19)	14.87 (SD 6.19)	15.13 (SD 8.24)	0.954

**Table 7:** Statistical correlations between GDM and blood biomarkers.

BIOMARKER (N)	GDM (N = 10): Mean (SD)–Median (IQR)	No GDM (N = 78): Mean (SD)–Median (IQR)	p-values
sVAP-1 (N = 10, 78)	366.73 (IQR 274.28–466.36)	428.34 (IQR 336.34–552.18)	0.127
NT (N = 9, 71)	0.95 (IQR 0.88–1.62)	1.01 (IQR 0.87–1.21)	0.711
Free beta hCG (N = 3, 10)	0.92 (IQR 0.67–1.41)	1.00 (IQR 0.68–1.67)	0.698
PAPP-A (N = 9, 70)	1.07 (IQR 0.76–1.4)	1.13 (IQR 0.73–1.54)	0.701
Hb at booking (N = 10, 78)	129.2 (SD 12.96)	129.88 (SD 10.57)	0.851
WCC at booking (N = 10, 78)	9.33 (SD 2.20)	9.16 (SD 2.38)	0.830
RBC at booking (N = 10, 78)	4.55 (SD 0.52)	4.37 (SD 0.36)	0.170
Hct at booking (N = 10, 78)	0.40 (SD 0.04)	0.40 (SD 0.03)	0.775
MCV at booking (N = 9, 78)	91 (IQR 90–94)	92 (IQR 88–95)	0.387
Platelets at booking (N = 10, 78)	259.8 (SD 63.91)	239.54 (SD 50.53)	0.250
Neutrophils (N = 10, 78)	6.35 (SD 2.08)	6.57 (SD 2.06)	0.755
Eosinophils at booking (N = 10, 78)	0.12 (IQR 0.1–0.24)	0.11 (IQR 0.07–0.21)	0.465
Basophils at booking (N = 9, 78)	0.03 (SD 0.01)	0.04 (SD 0.02)	0.081
Monocytes at booking (N = 10, 78)	0.54 (IQR 0.46–0.65)	0.45 (IQR 0.39–0.56)	0.216
Total lymphocytes at booking (N = 10, 78)	1.89 (IQR 1.75–2.55)	1.89 (IQR 1.54–2.21)	0.197
OGTT, test 1 (N = 6, 38)	4.95 (IQR 4.4–5.5)	4.2 (IQR 4.1–4.5)	0.004
OGTT, test 2 (N = 10, 38)	8.6 (IQR 8–10.7)	5.45 (IQR 4.6–6.4)	<0.001
Hb at 28 GW (N = 10, 77)	115.1 (SD 6.92)	117.75 (SD 10.33)	0.433
WCC at 28 GW (N = 10, 77)	12.1 (IQR 10.6–12.4)	9.9 (IQR 8.1–11.9)	0.050
RBC at 28 GW (N = 10, 77)	4.05 (SD 0.52)	3.94 (SD 0.37)	0.424
Hct at 28 GW (N = 10, 77)	0.34 (SD 0.02)	0.35 (SD 0.03)	0.481
MCV at 28 GW (N = 10, 77)	85.6 (SD 7.99)	80.51 (SD 6.25)	0.075
Platelets at 28 GW (N = 10, 77)	302.2 (SD 54.56)	262.93 (SD 63.90)	0.067
Neutrophils at 28 GW (N = 10, 77)	9.33 (IQR 7.68–9.93)	7.24 (IQR 5.79–9.13)	0.025
Eosinophils at 28 GW (N = 10, 77)	0.14 (IQR 0.11–0.17)	0.12 (IQR 0.08–0.18)	0.281
Basophils at 28 GW (N = 10, 77)	0.04 (IQR 0.02–0.04)	0.03 (IQR 0.02–0.04)	0.862
Monocytes at 28 GW (N = 10, 77)	0.50 (IQR 0.45–0.58)	0.49 (IQR 0.43–0.59)	0.930
Total lymphocytes at 28 GW (N = 10, 77)	1.91 (IQR 1.83–2.4)	1.75 (IQR 1.59–2.14)	0.049
ALT (N = 2, 18)	13 (IQR 12–14)	15 (IQR 14–17)	0.232
Albumin (N = 2, 21)	41 (SD 1.41)	41.81 (SD 3.14)	0.726
Total bilirubin (N = 2, 21)	4 (IQR 4–4)	6 (IQR 5–7)	0.119
Total protein (N = 2, 21)	64.5 (IQR 64–65)	68 (IQR 64–70)	0.356
Creatinine (N = 9, 22)	41 (IQR 40–42)	47.5 (IQR 42–50)	0.207
Potassium (N = 9, 23)	4.24 (SD 0.26)	4.00 (SD 0.37)	0.077
Sodium (N = 9, 23)	137 (IQR 135–138)	137 (IQR 136–138)	0.668
Urea (N = 9, 23)	3 (SD 0.63)	2.86 (SD 0.56)	0.546
HbA1c, % (N = 10, 14)	5.75 (IQR 5.2–6)	5.15 (IQR 5–5.3)	0.012
HbA1c (N = 10, 14)	38.1 (SD 5.38)	32.36 (SD 2.62)	0.002
Protein/creatinine ratio (N = 4, 14)	12.65 (IQR 10.3–14.65)	13.85 (IQR 12.2–21.9)	0.344
Random protein (N = 4, 15)	0.27 (IQR 0.25–0.30)	0.2 (IQR 0.15–0.34)	0.253
Random creatinine (N = 4, 19)	22.37 (SD 3.36)	13.55 (SD 7.62)	0.036

In contrast to that highlighted for PIH, sVAP-1 median levels did not differ in women carrying a growth restricted fetus as compared to the ones with fetuses with normal growth curves (424.32 ng/mL and 424.52 ng/mL, respectively) (Table 6). Thus, according to the results of our study, sVAP -1 is not a promising biomarker for FGR detection. As shown below, full blood count parameters in FGR were consistently lower than in the control population. PAPP-A measurements were also lower, while free-beta hCG appeared to be raised in the mothers of the small for gestational age fetuses (Table 6).

Interestingly, our study showed lower levels of sVAP-1 in women with GDM as compared to the normoglycemic study population (366.73 ng/mL vs 428.34 ng/mL, respectively), suggesting a potential role of sVAP-1 in predicting the onset of GDM in the second and third trimesters of pregnancy. As expected, both OGTT tests and glycated haemoglobin (HbA1c) were higher in women with GDM. Nuchal translucency (NT), free-βhCG, and PAPP-A are all decreased in GDM. A moderate state of lymphocytosis was detected in GDM as compared to control women when a diagnosis of GDM is made (24–28 GW). Platelets were moderately higher at both blood collection timepoints (booking and 28 GW) (Table 7).

Soluble VAP-1 levels in women who experienced a pregnancy complication different from those already analysed were slightly higher (446.35 ng/mL) than levels in women without any of these complications (424.42 ng/mL) (S3 Table). However, the difference (21.93 ng/mL) is not significant for sVAP-1 to be considered a useful diagnostic tool for the onset of generic adverse pregnancy outcomes. Besides a moderate increase in the total lymphocyte count at booking, no remarkable difference can be identified between women with or without one of the aforementioned pregnancy complications.

Serum levels of sVAP-1 in the healthy population (428.34 ng/mL) were not statistically significantly different from women with any of the complications considered in our study (421.28 ng/mL). In a similar way, none of the other biomarkers potentially useful for disease prediction (PAPP-A, βhCG) seem to differ enough between women experiencing complications and healthy study population (S4 Table).

## Discussion

The aim of this pilot study was to preliminary explore potential correlations existing between sVAP-1 serum measurements and pregnancy complications. Initially planned as a definitive study but then changed in view of interim findings, we believe that our study provides high quality pilot data for future studies, and this could enable future research to further investigate sVAP-1 potential as an early pregnancy biomarker of adverse pregnancy outcomes.

According to our results, sVAP-1 might be a promising early biomarker for the onset of PIH and GDM before the onset of clinical symptoms of disease (<20 GW). No association between VAP-1 and FGR emerged; this was probably due to the fact that no distinction was made between early and late-onset FGR, which are regarded to be two different diseases characterised by different aetiopathogeneses [28]. Because of a small study population, a separate individual statistical analysis for other pregnancy complications was not feasible, thus a plethora of different pregnancy diseases were grouped and analysed as a composite outcome. As a result, no difference in sVAP-1 levels were reported (Table 8).

**Table 8:** Summary of significant findings.

Pregnancy complication	Participants developing complication (N) / controls (N)	sVAP-1 serum levels in patients with complication (ng/mL)	sVAP-1 serum levels in controls (ng/mL)	sVAP-1 decreased (↓) or comparable (≈) in complication group
PIH	7 / 81	310.33 (IQR 242.68–433.56)	427.44 (IQR 347.38–552.18)	**↓**
FGR	7 / 81	424.32 (IQR 343.53–694.78)	424.52 (IQR 331.98–519.35)	**≈**
GDM	10 / 78	366.73 (IQR 274.28–466.36)	428.34 (IQR 336.34–552.18)	**↓**
Other complications	16 / 72	446.35 (IQR 335.88–667.76)	424.42 (IQR 333.13–517.95)	**≈**
Any complication	36 / 55	421.28 (IQR 300.10–589.44)	428.34 (IQR 341.88–525.76)	**≈**

As explored in several studies [16, 22, 29, 30], VAP-1 levels proved to be consistently associated with disease prediction or progression. However, current literature lacks data regarding VAP-1 and pregnancy. Indeed, to the best of our knowledge, our pilot work represents the second clinical study assessing VAP-1 levels in a pregnant population and the first one to focus on pregnancy hypertension. In order to reduce bias, all the blood samples were stored and analysed anonymously, and the researcher was unaware of the pregnancy outcomes of any of the samples before performing the analysis. Another strength of our study is the accurate extraction of individual patient data. Among the limitations beyond small population size, sVAP-1 levels have been prospectively measured using ELISA; all the other blood parameters were retrospectively collected via electronic hospital records.

The result of our study found an interesting association between lower sVAP-1 serum levels measured before 20 GW and subsequent onset of GDM at a later stage of gestation. This is in contrast with the only other VAP-1 and GDM study present in literature, which showed increased levels of VAP-1 in GDM compared to healthy controls [22]. However, Dincgez Cakmak et al. [22] assessed women at a later gestational age (24–28 GW), when a disease of GDM was already made. Furthermore, even if the sample size was similar to our study (135 patients), the study was not blinded, and samples were withdrawn from two specific groups of people (with or without GDM). Also, according to the study results [22], VAP-1 values were widely different from those reported in the literature, which appear to be in line with ours [31, 32]. Finally, a different ELISA kit was used [22].

In summary, our pilot study has validated the accuracy and reliability of our ELISA methodology using a commercially available ELISA kit in measuring circulating VAP-1 levels in early pregnancy, including the working range. The findings of our pilot study are indicative, however, crucially, will now allow us to design and power future studies to confirm the clinical utility of VAP-1 levels as an early pregnancy predictor of pregnancy complications.

## Supporting information

S1 Table Descriptive analysis and statistical tests by other pregnancy complications. (PDF)

S2 Table Descriptive analysis and statistical tests by all pregnancy complications. (PDF)

S3 Table Statistical correlation between other pregnancy complications and blood biomarkers. (PDF)

S4 Table Statistical correlation between all pregnancy complications and blood biomarkers. (PDF)
