# Supplementary material for: Soluble Vascular Adhesion Protein 1 (sVAP-1) as a biomarker for pregnancy complications: A pilot study
Source: PLoS One. 2023 May 30;18(5):e0284412. doi: 10.1371/journal.pone.0284412 (PMC10228776; doi:10.1371/journal.pone.0284412)
Supplement: S1 Table — (PDF) [file pone.0284412.s001.pdf]

S1 Table. Descriptive analysis and statistical tests by other pregnancy complications.

VARIABLES (N)	Other pregnancy complication (N=16): Mean (SD) – Median (IQR) – N(%)	No other pregnancy complication (N=72): Mean (SD) – Median (IQR) – N(%)	p-values
AGE (N=81)	29.81 (SD 5.69)	29.49 (5.09)	0.821
BMI (N=89)	24.24 (IQR 21.67-26.17)	24.77 (IQR 21.88-29.92)	0.415
BLOOD TYPE (N=90)			
0	7 (43.75)	26 (36.62%)	0.828
A	7 (43.75)	30 (42.25%)	
B	2 (12.50%)	13 (18.31%)	
AB	0 (0%)	2 (2.82%)	
BLOOD RHESUS (N=90)			
Rh +	14 (87.50%)	60 (84.51%)	0.762
Rh -	2 (12.50%)	11 (15.49%)	
GRAVIDITY (N=88)			
G1	5 (33.33%)	17 (24.29%)	0.047
G2	5 (33.33%)	32 (32.86%)	
G3	1 (6.67%)	9 (12.86%)	
G4	0 (0%)	5 (7.14%)	
G5	0 (0%)	11 (15.71%)	
G6	1 (6.67%)	1 (1.43%)	
G7	0 (0%)	2 (2.86%)	
G8	1 (6.67%)	1 (1.43%)	
G9	2 (13.33%)	0 (0%)	
G10	0 (0%)	1 (1.43%)	

PARITY (N=88)			
P0	5 (33.33%)	24 (34.29%)	0.433
P1	6 (40%)	23 (32.86%)	
P2	0 (0%)	13 (18.57%)	
P3	1 (6.67%)	4 (5.71%)	
P4	2 (13.33%)	3 (4.29%)	
P5	1 (6.67%)	3 (4.29%)	
MOTHER ETHNICITY (N=91)			
White	13 (81.25%)	56 (77.78%)	0.956
Asian	2 (12.50%)	11 (15.28%)	
Black	1 (6.25%)	4 (5.56%)	
Others	0 (0%)	1 (1.29%)	
BABY'S FATHER ETHNICITY (N=91)			
White	11 (68.75%)	48 (66.67%)	0.972
Asian	3 (18.75%)	15 (20.83%)	
Black	1 (6.25%)	6 (8.33%)	
Others	1 (6.25%)	3 (4.17%)	
SMOKING (N=91)			
No = 10 (62.50%)	10 (62.50%)	42 (58.33%)	0.620
Yes (cigarettes) = 2 (12.50%)	2 (12.50%)	14 (19.44%)	
Yes (e-cigarette) = 0 (0%)	0 (0%)	4 (5.56%)	
Yes, but stopped	4 (25%)	12 (16.67%)	
SMOKERS IN HOUSEHOLD (N=91)			
No	11 (68.75%)	55 (76.39%)	0.523
Yes	5 (31.25%)	17 (23.61%)	

ALCOHOL/SUBSTANCES ABUSE (N=91)			
No	10 (62.50%)	45 (62.50%)	0.951
Stopped alcohol	5 (31.25%)	23 (31.94%)	
Stopped drugs	0 (0%)	1 (1.39%)	
Yes	1 (6.25%)	3 (4.17%)	
THROMBOEMBOLIC RISK (N=91)			
Low	11 (68.75%)	54 (75%)	0.125
Intermediate	0 (0%)	8 (11.11%)	
High	5 (31.25%)	10 (13.89%)	
PREGNANCY CATEGORY OF RISK (N=91)			
Low	5 (31.25%)	35 (48.61%)	0.207
High	11 (68.75%)	37 (51.39%)	
FERTILITY TREATMENT (N=91)			
No	15 (93.75%)	70 (97.22%)	0.489
Yes	1 (6.25%)	2 (2.78%)	
PLANNED PREGNANCY (N=91)			
No	8 (50%)	26 (36.11%)	0.391
Yes	8 (50%)	41 (56.94%)	
No (on pill during conception)	0 (0%)	5 (6.94%)	
OUTCOME OF PAST PREGNANCIES (N=87)			0.853
Never delivered (P0 or TOP)	5 (33.33%)	19 (27.54%)	
Livebirths (P1+)	6 (40%)	27 (39.13%)	
Miscarriages, stillbirths, molar pregnancies	4 (26.67%)	23 (33.33%)	

NUMBER OF MAU ADMISSIONS (N=89)			
0	8 (50%)	30 (41.67%)	0.051
1	2 (12.5%)	19 (26.93%)	
2	1 (6.25%)	11 (15.28%)	
3	1 (6.25%)	3 (4.17%)	
4	2 (12.5%)	2 (2.78%)	
5	0 (0%)	4 (5.56%)	
6	2 (12.5%)	0 (0%)	
7	0 (0%)	2 (2.78%)	
8	0 (0%)	1 (1.39%)	
GW DELIVERY (N=88)			
Term	12 (75%)	67 (93.06%)	0.046
Pre-term	4 (25%)	4 (5.56%)	
Post-term	0 (0%)	1 (1.39%)	
EBL (N=88)	400 (IQR 250-500)	300 (IQR 200-500)	0.330
INFANT WEIGHT (N=87)	3090 (IQR 2860-3600)	3430 (IQR 2040-3770)	0.216
INFANT SEX (N=86)			
Female	7 (50%)	25 (25 (34.72%)	0.297
Male	7 (50%)	47 (65.28%)	
INFANT OUTCOME (N=88)			
Livebirth	14 (87.50%)	72 (100%)	0.002
Miscarriage / TOP	2 (12.50%)	0 (0%)	
