# Supplementary material for: Soluble Vascular Adhesion Protein 1 (sVAP-1) as a biomarker for pregnancy complications: A pilot study
Source: PLoS One. 2023 May 30;18(5):e0284412. doi: 10.1371/journal.pone.0284412 (PMC10228776; doi:10.1371/journal.pone.0284412)
Supplement: S2 Table — (PDF) [file pone.0284412.s002.pdf]

S2 Table. Descriptive analysis and statistical tests by all complications.

VARIABLES (N)	Any pregnancy complication (N=36): Mean (SD) – Median (IQR) – N(%)	No pregnancy complication (N=55): Mean (SD) – Median (IQR) – N(%)	p-values
AGE (N=91)	29.03 (SD 4.84)	29.90 (5.41)	0.438
BMI (N=89)	25.93 (IQR 22.18-31.55)	24.50 (IQR 21.66-27.91)	0.193
BLOOD TYPE (N=90)			0.683
0	13 (37.14%)	20 (38.46%)	
A	16 (45.71%)	21 (40.38%)	
B	6 (17.14%)	9 (17.31%)	
AB	0 (0%)	2 (3.85%)	
BLOOD RHESUS (N=90)			
Rh +	33 (94.29%)	41 (78.85%)	0.048
Rh -	2 (5.71%)	11 (21.15%)	
GRAVIDITY (N=88)			
G1	9 (25.71%)	13 (26%)	0.503
G2	10 (28.57%)	18 (36%)	
G3	4 (11.43%)	6 (12%)	
G4	2 (5.71%)	3 (6%)	
G5	5 (14.29%)	5 (12%)	
G6	1 (2.86%)	1 (2%)	
G7	0 (0%)	2 (4%)	
G8	2 (5.71%)	0 (0%)	
G9	2 (5.71%)	0 (0%)	
G10	0 (0%)	1 (2%)	
PARITY (N=88)			

P0	11 (31.43%)	18 (36%)	0.715
P1	11 (31.43%)	18 (36%)	
P2	7 (20%)	6 (12%)	
P3	1 (2.86%)	4 (8%)	
P4	3 (8.57%)	2 (4%)	
P5	2 (5.71%)	2 (4%)	
MOTHER ETHNICITY (N=91)			
White	27 (75%)	42 (80.77%)	0.640
Asian	6 (16.67%)	7 (13.46%)	
Black	2 (5.56%)	3 (5.77%)	
Others	1 (2.78%)	0 (0%)	
BABY’S FATHER ETHNICITY (N=91)			
White	24 (66.67%)	35 (67.31%)	0.880
Asian	8 (22.22%)	10 (19.23%)	
Black	2 (5.56%)	5 (9.62%)	
Others	2 (5.56%)	2 (3.85)	
SMOKING (N=91)			
No	22 (61.11%)	30 (57.69%)	0.296
Yes (cigarettes)	4 (11.11%)	12 (23.08%)	
Yes (e-cigarette)	1 (2.78%)	3 (5.77%)	
Yes, but stopped	9 (25%)	7 (13.46%)	
SMOKERS IN HOUSEHOLD (N=91)			
No	25 (69.44%)	41 (78.85%)	0.317
Yes	11 (30.56%)	11 (21.15%)	
ALCOHOL/SUBSTANCES ABUSE (N=91)			

No	25 (69.44%)	30 (57.69%)	0.595
Stopped alcohol	10 (27.78%)	18 (34.62%)	
Stopped drugs	0 (0%)	1 (1.92%)	
Yes	1 (2.78%)	3 (5.77%)	
THROMBOEMBOLIC RISK (N=91)			0.068
Low	24 (66.67%)	41 (78.85%)	
Intermediate	2 (5.56%)	6 (11.54%)	
High	10 (27.78%)	5 (9.62%)	
PREGNANCY CATHEGORY OF RISK (N=91)			
Low	10 (27.78%)	30 (57.69%)	0.006
High	26 (72.22%)	22 (42.31%)	
FERTILITY TREATMENT (N=91)			
No	35 (97.22%)	50 (96.15%)	0.786
Yes	1 (2.78%)	2 (3.85%)	
PLANNED PREGNANCY (N=91)			
No	13 (36.11%)	31 (40.38%)	0.652
Yes	20 (55.56%)	29 (55.77%)	
No (on pill during conception)	3 (8.33%)	2 (3.85%)	
OUTCOME OF PAST PREGNANCIES (N=87)			
Never delivered (P0 or TOP)	9 (25.71%)	15 (30.61%)	0.426
Livebirths (P1+)	12 (34.29%)	21 (47.86%)	
Miscarriages, stillbirths, molar pregnancies	14 (40%)	13 (26.53%)	
NUMBER OF MAU ADMISSIONS (N=89)			
0	13 (36.11%)	25 (48.08%)	0.468
1	8 (22.22%)	13 (25%)	

2<br>3<br>4<br>5<br>6<br>7<br>8	4 (11.11%)<br>2 (5.56%)<br>3 (8.33%)<br>2 (5.56%)<br>2 (5.56%)<br>1 (2.78%)<br>1 (2.78%)	8 (15.38%)<br>2 (3.85%)<br>1 (1.92%)<br>2 (3.85%)<br>0 (0%)<br>1 (1.92%)<br>0 (0%)	
GW DELIVERY (N=88)<br>Term<br>Pre-term<br>Post-term	 28 (77.78%)<br>8 (22.22%)<br>0 (0%)	 51 (98.08%)<br>0 (0%)<br>1 (1.92%)	 0.001
EBL (N=88)	300 (IQR 200-475)	332.5 (IQR 225-500)	0.515
INFANT WEIGHT (N=87)	3090 (IQR 2600-3600)	3500 (IQR 3190-3855)	0.004
INFANT SEX (N=86)<br>Female<br>Male	 12 (35.29%)<br>22 (64.71%)	 20 (38.46%)<br>32 (61.54%)	 0.766
INFANT OUTCOME (N=88)<br>Livebirth<br>Miscarriage / TOP	 34 (94.44%)<br>2 (5.56%)	 52 (100%)<br>0 (0%)	 0.086
