# Supplementary material for: Soluble Vascular Adhesion Protein 1 (sVAP-1) as a biomarker for pregnancy complications: A pilot study
Source: PLoS One. 2023 May 30;18(5):e0284412. doi: 10.1371/journal.pone.0284412 (PMC10228776; doi:10.1371/journal.pone.0284412)
Supplement: S3 Table — (PDF) [file pone.0284412.s003.pdf]

Table S13. Statistical correlations between other pregnancy complications and blood biomarkers.

BIOMARKER (N)	Other pregnancy complications (N=16): Mean (SD) – Median (IQR) – N(%)	No other pregnancy complication (N=72): Mean (SD) – Median (IQR) – N(%)	p-values
sVAP-1 (N = 16, 72)	446.35 (IQR 335.88-667.76)	424.42 (IQR 333.13-517.95)	0.475
NT (N = 16, 64)	1.02 (IQR 0.95-1.3)	1 (IQR 0.86-1.89)	0.429
Free beta hCG (N = 16, 70)	0.98 (IQR 0.79-1.68)	1 (IQR 0.64-1.55)	0.739
PAPP-A (N 16, 63)	1.36 (IQR 1-1.71)	1.11 (IQR 0.68-1.49)	0.143
Hb at booking (N = 16, 72)	131.25 (SD 10.55)	129.49 (SD 10.88)	0.557
WCC at booking (N = 16, 72)	9.16 (SD 1.81)	9.18 (SD 2.47)	0.967
RBC at booking (N = 16, 72)	4.49 (SD 0.19)	4.37 (SD 0.40)	0.274
Hct at booking (N = 16, 72)	0.40 (SD 0.03)	0.40 (SD 0.03)	0.739
MCV at booking (N = 16, 72)	88.5 (IQR 84-92)	93 (IQR 89-95)	0.022
Platelets at booking (N = 16, 72)	262.5 (SD 59.16)	237.25 (SD 49.81)	0.080
Neutrophils at booking (N = 16, 72)	6.54 (SD 1.52)	6.54 (SD 2.16)	1.000
Eosinophils at booking (N = 16, 72)	0.13 (IQR 0.08-0.23)	0.11 (IQR 0.07-0.20)	0.365
Basophils at booking (N = 16, 72)	0.04 (SD 0.02)	0.04 (SD 0.02)	0.720
Monocytes at booking (N = 16, 72)	0.49 (IQR 0.38-0.59)	0.47 (IQR 0.4-0.57)	0.774
Total lymphocytes at booking (N = 16, 72)	1.94 (IQR 1.60-2.24)	1.89 (IQR 1.58-2.22)	0.750
OGTT, test 1 (N = 5, 43)	4.8 (IQR 4.1-5.4)	4.3 (IQR 4.1-4.5)	0.515
OGTT, test 2 (N = 5, 43)	5.4 (IQR 4.5-6.6)	6.1 (IQR 4.9-7.4)	0.434
Hb at 28 GW (N = 15, 72)	117.33 (SD 11.39)	117.47 (SD 9.78)	0.961
WCC at 28 GW (N = 15, 72)	8.5 (IQR 6.1-11.6)	10.4 (IQR 8.65-12.35)	0.053
RBC at 28 GW (N = 15, 72)	4.04 (SD 0.27)	3.94 (SD 0.41)	0.382

Hct at 28 GW (N = 15, 72)	0.35 (SD 0.03)	0.35 (SD 0.03)	0.851
MCV at 28 GW (N = 15, 72)	87.67 (SD 6.66)	89.35 (SD 6.52)	0.368
Platelets at 28 GW (N = 15, 72)	266 (SD 75.52)	267.75 (SD 61.76)	0.924
Neutrophils at 28 GW (N = 15, 72)	6.39 (IQR 4.81-9.21)	7.91 (IQR 6.27-9.51)	0.111
Eosinophils at 28 GW (N = 15, 72)	0.1 (IQR 0.06-0.2)	0.12 (IQR 0.09-0.18)	0.338
Basophils at 28 GW (N = 15, 72)	0.03 (IQR 0.02-0.05)	0.03 (IQR 0.02-0.04)	0.412
Monocytes at 28 GW (N = 15, 72)	0.49 (IQR 0.42-0.56)	0.49 (IQR 0.43-0.59)	0.900
Total lymphocytes at 28 GW (N = 15, 72)	1.66 (IQR 1.36-2.14)	1.86 (IQR 1.61-2.24)	0.237
ALT (N = 5, 15)	15 (IQR 14-17)	15 (IQR 14-17)	0.962
Albumin (N = 6, 17)	41.67 (SD 3.50)	41.76 (SD 2.95)	0.947
ALP (N = 6, 16)	96.17 (SD 45.14)	84.56 (SD 26.19)	0.458
Total bilirubin (N = 6, 17)	6 (IQR 5-7)	6 (IQR 4-7)	0.776
Total protein (N = 6, 17)	68.5 (IQR 63-70)	67 (IQR 64-70)	0.773
Creatinine (N = 8, 23)	43.5 (IQR 39.5-44.5)	46 (IQR 40-51)	0.332
Potassium (N = 8, 24)	3.92 (SD 0.26)	4.11 (SD 0.38)	0.206
Sodium (N = 8, 24)	136.5 (IQR 135-138)	137 (IQR 135.5-138)	0.870
Urea (N = 8, 24)	2.89 (SD 0.56)	2.90 (SD 0.59)	0.945
HbA1c, % (N = 3, 21)	5 (IQR 4.6-5.3)	5.2 (IQR 5.1-5.6)	0.182
HbA1c (N = 3, 21)	31 (SD 3.61)	35.29 (SD 4.84)	0.157
Protein/creatinine ratio (N = 4, 14)	12.3 (IQR 11.9-20.9)	14.05 (IQR 11.3-16.9)	0.703
Random protein (N = 4, 15)	0.20 (IQR 0.17-0.27)	0.25 (IQR 0.19-0.3)	0.687
Random creatinine (N = 5, 18)	13.4 (SD 10.47)	15.57 (SD 7.19)	0.583
