# Supplementary material for: Soluble Vascular Adhesion Protein 1 (sVAP-1) as a biomarker for pregnancy complications: A pilot study
Source: PLoS One. 2023 May 30;18(5):e0284412. doi: 10.1371/journal.pone.0284412 (PMC10228776; doi:10.1371/journal.pone.0284412)
Supplement: S4 Table — (PDF) [file pone.0284412.s004.pdf]

S4 Table. Statistical correlations between all pregnancy complications and blood biomarkers.

**BIOMARKER (N)**	**Any pregnancy complication (N=36): Mean (SD) – Median (IQR) – N(%)**	**No pregnancy complication (N=55): Mean (SD) – Median (IQR) – N(%)**	p-values
sVAP-1 (N = 36, 52)	421.28 (IQR 300.10-589.44)	428.34 (IQR 341.88-525.76)	0.585
NT (N = 34, 46)	1.01 (IQR 0.9-1.33)	1 (IQR 0.84-1.13)	0.161
Free beta hCG (N = 36, 50)	0.98 (IQR 0.68-1.48)	1.06 (IQR 0.64-1.72)	0.684
PAPP-A (N = 34, 45)	1.14 (IQR 0.76-1.54)	1.11 (IQR 0.68-1.49)	0.758
Hb at booking (N = 36, 52)	131.19 (SD 12.45)	128.85 (SD 9.47)	0.318
WCC at booking (N = 36, 52)	9.01 (SD 2.11)	9.29 (SD 2.52)	0.588
RBC at booking (N = 36, 52)	4.48 (SD 0.39)	4.34 (SD 0.37)	0.093
Hct at booking (N = 36, 52)	0.40 (SD 0.03)	0.40 (SD 0.03)	0.578
MCV at booking (N = 36, 52)	91 (IQR 86-94)	93 (IQR 89-95.5)	0.115
Platelets at booking (N = 36, 52)	247.28 (SD 61.28)	238.08 (SD 45.11)	0.419
Neutrophils at booking (N = 36, 52)	6.34 (SD 1.83)	6.69 (SD 2.20)	0.434
Eosinophils at booking (N = 36, 52)	0.11 (IQR 0.07-0.21)	0.11 (IQR 0.07-0.21)	0.924
Basophils at booking (N = 35, 52)	0.04 (SD 0.02)	0.04 (SD 0.02)	0.069
Monocytes at booking (N = 36, 52)	0.49 (IQR 0.40-0.58)	0.45 (IQR 0.40-0.57)	0.687
Total lymphocytes at booking (N = 36, 52)	1.93 (IQR 1.67-2.26)	1.87 (IQR 1.51-2.18)	0.239
OGTT, test 1 (N = 20, 28)	4.5 (IQR 4.1-5.25)	4.3 (IQR 4.1-4.5)	0.117
OGTT, test 2 (N = 20, 28)	7.25 (IQR 5.3-8.6)	5.65 (IQR 4.75-6.35)	0.011
Hb at 28 GW (N = 35, 52)	118.51 (SD 11.14)	116.73 (SD 9.20)	0.418
WCC at 28 GW (N = 35, 52)	10.4 (IQR 7.4-12.2)	10 (IQR 8.35-12.35)	0.476
RBC at 28 GW (N = 35, 52)	4.02 (SD 0.38)	3.91 (SD 0.39)	0.204
Hct at 28 GW (N = 35, 52)	0.36 (SD 0.03)	0.35 (SD 0.03)	0.375

MCV at 28 GW (N = 35, 52)	88.63 (SD 7.50)	89.35 (SD 5.87)	0.619
Platelets at 28 GW (N = 35, 52)	267.83 (SD 67.95)	267.19 (SD 61.63)	0.964
Neutrophils at 28 GW (N = 35, 52)	7.68 (IQR 5.28-9.25)	7.81 (IQR 6.22-9.34)	0.851
Eosinophils at 28 GW (N = 35, 52)	0.13 (IQR 0.07-0.18)	0.12 (IQR 0.09-0.19)	0.728
Basophils at 28 GW (N = 35, 52)	0.03 (IQR 0.02-0.04)	0.03 (IQR 0.03-0.05)	0.111
Monocytes at 28 GW (N = 35, 52)	0.49 (IQR 0.4-0.55)	0.5 (IQR 0.44-0.61)	0.148
Total lymphocytes at 28 GW (N = 35, 52)	1.83 (IQR 1.61-2.14)	1.82 (IQR 1.6-2.18)	0.808
ALT (N = 12, 8)	15 (IQR 13-17)	15 (IQR 14-16.5)	0.802
Albumin (N = 13, 10)	41.46 (SD 2.88)	42.1 (SD 3.31)	0.626
ALP (N = 12, 10)	96.25 (SD 38.60)	77.5 (SD 17.53)	0.172
Total bilirubin (N = 13, 10)	6 (IQR 5-7)	5.5 (IQR 4-7)	0.544
Total protein (N = 13, 10)	66 (IQR 64-70)	68 (IQR 66-70)	0.681
Creatinine (N = 21, 10)	43 (IQR 40-49)	46.5 (IQR 34-51)	0.610
Potassium (N = 21, 11)	4.11 (SD 0.31)	3.98 (SD 0.45)	0.347
Sodium (N = 21, 11)	137 (IQR 136-138)	137 (IQR 135-138)	0.864
Urea (N = 21, 11)	2.88 (SD 0.60)	2.95 (SD 0.55)	0.751
HbA1c, % (N = 14, 10)	5.25 (IQR 5.21-6)	5.15 (IQR 5-5.3)	0.131
HbA1c (N = 14, 10)	36.21 (SD 5.65)	32.7 (SD 2.50)	0.080
Protein/creatinine ratio (N = 13, 5)	13.6 (IQR 12.2-15.3)	14.1 (IQR 10-21.9)	1.000
Random protein (N = 13, 6)	0.25 (IQR 0.19-0.29)	0.20 (IQR 0.13-0.47)	0.813
Random creatinine (N = 14, 9)	15.83 (SD 8.28)	13.93 (SD 7.29)	0.581
